# Incorporation of Nanostructural Hydroxyapatite and Curcumin Extract from *Curcuma longa* L. Rhizome into Polylactide to Obtain Green Composite

**DOI:** 10.3390/polym16152169

**Published:** 2024-07-30

**Authors:** Magdalena Osial, Sławomir Wilczewski, Urszula Godlewska, Katarzyna Skórczewska, Jakub Hilus, Joanna Szulc, Agata Roszkiewicz, Agnieszka Dąbrowska, Zahra Moazzami Goudarzi, Krzysztof Lewandowski, Tomasz P. Wypych, Phuong Thu Nguyen, Grzegorz Sumara, Michael Giersig

**Affiliations:** 1 Institute of Fundamental Technological Research, Polish Academy of Sciences, Pawińskiego 5B Str., 02-106 Warsaw, Poland; arosz@ippt.pan.pl (A.R.); zahramoazami8@gmail.com (Z.M.G.); mgiersig@ippt.pan.pl (M.G.); 2 Faculty of Chemical Technology and Engineering, Bydgoszcz University of Science and Technology, Seminaryjna 3 Str., 85-326 Bydgoszcz, Poland; katarzyna.skorczewska@pbs.edu.pl (K.S.); joanna.szulc@pbs.edu.pl (J.S.); krzysztof.lewandowski@pbs.edu.pl (K.L.); 3 Laboratory of Host-Microbiota Interactions, Nencki Institute of Experimental Biology, Polish Academy of Sciences, Pasteura 3 Str., 02-093 Warsaw, Poland; u.godlewska@ujd.edu.pl (U.G.); t.wypych@nencki.edu.pl (T.P.W.); 4 Collegium Medicum, Jan Długosz University in Czestochowa, 13/15 Armii Krajowej Str., 42-200 Czestochowa, Poland; 5 Faculty of Medicine, Jagiellonian University Medical College, St Anne 12 Str., 31-008 Cracow, Poland; jakub.hilus@student.uj.edu.pl; 6 Faculty of Chemistry, University of Warsaw, Pasteura 1 Str., 02-093 Warsaw, Poland; adabrowska@chem.uw.edu.pl; 7 Institute for Tropical Technology, Vietnam Academy of Science and Technology, 18 Hoang Quoc Viet, Cau Giay, Hanoi 100000, Vietnam; ntphuong@itt.vast.vn; 8 Dioscuri Centre for Metabolic Diseases, Nencki Institute of Experimental Biology PAS, 02-093 Warsaw, Poland; g.sumara@nencki.edu.pl

**Keywords:** polylactide, hydroxyapatite, turmeric extract, curcumin extract, green composite

## Abstract

This study showed that a polylactide (PLA)-based composite filled with nanostructured hydroxyapatite (HAp) and a natural extract from the rhizome of *Curcuma longa* L. could provide an alternative to commonly used fossil-based plasticsfor food packaging. The incorporation of HAp into the PLA matrix had a positive effect on improving selected properties of the composites; the beneficial effect could be enhanced by introducing a green modifier in the form of an extract. Prior to the fabrication of the composite, the filler was characterized in terms of morphology and composition, and the composite was then fully characterized by scanning electron microscopy (SEM), atomic force microscopy (AFM), Raman and Fourier transform infrared spectroscopy (FT-IR), and the mechanical, thermal, thermomechanical, and optical properties were investigated. The proposed material exhibits antioxidant properties against DPPH radicals and antibacterial performance against *Escherichia coli* (*E. coli*). The results showed that the nanocomposite has the highest antioxidant and antibacterial properties for 10 wt% HAp with an average diameter of rod-shaped structures below 100 nm. In addition, the introduction of turmeric extract had a positive effect on the tensile strength of the nanocomposites containing 1 and 5% HAp. As the resulting material adsorbs light in a specific wavelength range, it can be used in the medical sector, food-packaging, or coatings.

## 1. Introduction

Polylactide (PLA) has gained popularity in various fields due to its biodegradability and good mechanical properties similar to synthetic polymers such as polystyrene. Its properties make it suitable for use in textiles, packaging, and biomedical applications. Therefore, different renewable resources, such as maize starch and sugar cane, are widely used to produce PLA. Its synthesis can be easily performed from lactic acid by direct polycondensation or ring-opening polymerization, making it cost-effective and easy to produce [1,2,3,4]. As pure PLA has limited applications, physical and chemical modifications are introduced to improve its properties and thus increase its application areas. A good example is the modification of end groups, which enables increased control of the polymer’s hydrolysis, and ensures better thermal properties, and improves its self-organization and interaction with nanoparticles or metal ions [5,6]. In addition to modifying the polymer itself, a number of studies show the advantages of adding fillers to PLA to modify its properties [7,8,9] such as thermomechanical properties [10,11], or resistance to aging [12], making it a promising platform to be used in many fields from biomedicine to industrial applications [13,14].

Among numerous fillers, hydroxyapatite (HAp), a ceramic bioactive and biocompatible material, offers biocompatibility and cost-effective production, making it a promising polymer modifier. HAp is a natural component of bone that provides density and mechanical resistance, which can be easily achieved due to its high similarity to natural apatite and high active surface area. It is composed of calcium and phosphorus and can be synthesized, for example, by precipitation using calcium hydroxide and phosphoric acid, maintaining a molar ratio of Ca/P = 1.67 like natural HAp. However, depending on the synthesis method used, it is possible to control its porosity, particle size, and morphology to use it as a filler in polymer-based composites [15,16,17,18,19]. Also, the shape memory effect can be obtained.

Recent studies have shown the potential of using polylactide matrix nanocomposites with nanohydroxyapatite via various methods, such as solvent casting and direct melt mixing [20]. The use of nanohydroxyapatite in PLA/HAp nanocomposites can improve the mechanical properties of these materials compared to the unmodified matrix material. The materials showed increased tensile strength, compressive strength, Young’s modulus, elongation at break, and improved dynamic mechanical properties compared to the unfilled polymer matrix. Yet, the improvement in mechanical properties depends on factors such as the number of defects in the structure of the HAp used, its content and degree of dispersion in the nanocomposites, and the favorable interactions at the filler–polymer interface [20,21,22,23]. The addition of nanohydroxyapatite to PLA can affect the thermal stability of the polymer. It has been observed that the thermal stability of nanocomposites can both increase and decrease, which depends on the filler content in the matrix and the specific surface area of the HAp used [21,24,25,26]. Besides the mechanical or thermal properties, numerous in vitro studies show excellent biological activity and biocompatibility of PLA/HAp nanocomposites, indicating that these materials are suitable for bone grafting and tissue engineering applications [20,23,27].

Due to the fact that PLA is a plastic formed from natural products that are suitable for composting, it is a promising material for application in many sectors including food-packaging. However, due to its high permeability to gases, it is not suitable for packaging selected food products [28]. In order to improve the barrier and mechanical properties of PLA in food packaging applications, a number of efforts have been made, such as combining it with cellulose nanocrystals [29], lactic acid-based ester precursors [30], reactive hardening agents [31], and by-products from the agro-food industry [32].

Summarizing the analysis of the available literature, it can be concluded that both PLA and HAp are interesting biocompatible materials that can be used in biomedical engineering and packaging. On the other hand, PLA/HAp nanocomposites may exhibit better mechanical and/or thermal properties compared to the unmodified polymer. The beneficial effect of the filler depends, in particular, on its content and dispersion in the matrix. Furthermore, it is highly desirable to use natural substances as an alternative to synthetic compounds to improve the dispersion of nanofillers and material’s flexibility.

As there is a deep need to deliver environmentally friendly materials to substitute plastics and reduce microplastic pollution globally [33], we aimed to deliver a biodegradable composite that is fully based on natural ingredients. The literature demonstrates the incorporation of curcuminoids into PLA [34] but for now, no studies have been found on the use of curcuminoids (CUs) to improve HAp dispersion in a PLA matrix. However, it is known that modification of HAp with CU can be useful in various applications, such as drug delivery, action against biofilm formation, and bone regeneration [35,36,37,38]. Taking this into account, the aim of the presented work was to produce a new green nanocomposite as an alternative to the fossil-derived plastics and fillers that can release many harmful compounds. An environmentally friendly composite based on polylactide and nanohydroxyapatite coated with an antibacterial plant extract from the rhizome of *Curcuma longa* L. as a natural, non-cytotoxic stabilizer and coloring agent, is proposed.

## 2. Materials and Methods

### 2.1. Materials

Calcium (II) nitrate tetrahydrate Ca(NO_3_)_2_·4H_2_O, diammonia monohydrophosphate (NH_4_)_2_HPO_4_, and sodium hydroxide NaOH, all with analytical grade, were supplied from CHEMPUR, Poland. Deionized water was obtained from the HYDROLAB water filtering system, Gliwice, Poland. Methanol (MeOH) (96%) and chloroform (98.5%) were purchased from POCH, Gliwice, Poland. Polylactide 2003D (PLA) granules were supplied from NatureWorks LLC. Minnetonka, MN, USA, with a density of 1240 kg m^−3^ and melt flow index of 5.92 g/10 min (190 °C/2.16 kg). *Curcuma longa* L. rhizome extract (CE) was obtained as in our earlier work [39]. Lysogeni broth (LB) and phosphate buffer saline (PBS) were supplied from Sigma Aldrich, Munich, Germany.

### 2.2. Hydroxyapatite Synthesis

Nanostructured hydroxyapatite was synthesized using a wet co-precipitation method based on our previous protocols [37], in which the synthesis was adjusted to yield approximately 1.76 g of HAp with one set of co-precipitation. To prepare HAp of the desired shape and size, 2.36 g of Ca(NO_3_)_2_ was dissolved in 25 mL of distilled water and stirred magnetically at a stirring speed of 600 rpm. Then, 25 mL of the solution containing 1.32 g of (NH_4_)_2_HPO_4_ was placed in a burette over the calcium-based solution, and the phosphates were added dropwise with 1 M NaOH from a second burette, where the NaOH was added at the same time as the phosphates to control the pH at 11. The pH was then controlled for 15 min, and the suspension was stirred continuously for 1 h. After this time, the HAp was washed with spilled water and dried in an oven overnight at 80 °C in a beaker. The dried HAp was then pulverized using a grinder. Next, powdered HAp was added to polylactide using the procedure described in Section 2.4. Nevertheless, as HAp has hydrophilic properties, the uniform distribution of the HAp into the polylactide was performed using an ultrasonic homogenizer for the bare HAp and HAp coated with a plant extract that enhances the distribution of the filler into the PLA matrix.

### 2.3. Hydroxyapatite Modification with Turmeric Extract

To modify the HAp (freshly obtained with the same procedure as described above and washed), it was placed into a beaker to sediment, and then the water was replaced with methanolic turmeric extract. The beaker was placed into an ultrasonic bath for 30 min and then the suspension was stirred overnight at 600 rpm. After that time, the filler was separated from the solution with centrifuging and dried in an oven for 3 h at 40 °C (a higher temperature could degrade the plant extract) to remove methanol. The coating was around ~10% wt. over HAp. Dried samples were grinded the same way as bare HAp for subsequent incorporation into the polylactide matrix.

### 2.4. Composites Preparation

In the first stage of preparation of the nanocomposites, polylactide was dissolved in chloroform at 25 °C for ca. 48 h, yielding a solution with a concentration of 7 wt.% Next, HAp and HAp@CE were added to the solution and dispersed for 60 min at 20 °C. The dispersion was enhanced ultrasonically with a frequency of 20 kHz and 40% amplitude, using a SONOPULS rod-shaped probe homogenizer from Bandelin, Berlin, Germany. The amount of the filler in the prepared dispersions was 1%, 5%, 10%, and 20% over polymer weight. Then, films of PLA/HAp nanocomposites were obtained by the solvent evaporation method onto Petri dishes with a diameter of 12 cm, where the solvent was evaporated at 40 °C for 24 h. The film was dried in a vacuum drier under reduced pressure (max 20 mbar absolute) at 40 °C for 2 weeks to remove the solvent residue. All samples were coded to take into account the HAp content and the presence of curcumin; for example, a sample containing only HAp at a concentration of 1, 5, 10, and 20 wt.% was named PLA/1HAp, PLA/5HAp, PLA/10HAp, and PLA/20HAp versus a sample containing the same filler content and curcumin extract, named PLA/1HAp@CE, PLA/5HAp@CE, PLA/10HAp@CE, and PLA/20HAp@CE, respectively.

### 2.5. Characterization Methods

The morphology of the materials was investigated using scanning electron microscopy (SEM) (Merlin, ZEISS, Stuttgart, Germany) where the samples were placed on an aluminum sample holder and immobilized with carbon conducting tape onto the alumina sample holder and transmission electron microscopy (TEM) (Zeiss Libra 120 Plus, Stuttgart, Germany) was performed, operating at 120 kV, where the HAp colloidal suspension was placed on the copper mesh covered with Formvar and dried.

The structure of composites was studied using the scanning electron microscopy (SEM) (Merlin, ZEISS, Stuttgart, Germany). The samples for SEM observation were fractured cryogenically and sputtered with a gold nanometric layer.

The larger-scale topography of the PLA and PLA-based composite with HAp and CE samples was examined with the Atomic Force Microscope (AFM) alpha 300 S (WITec GmbH, Ulm, Germany). An Al-coated AFM probe with a force constant of 0.2 N m^−1^ was used in the contact mode. The position of the probe with respect to the sample surface was controlled by an optical feedback system.

A light transmission test was performed on the film samples in the UV and visible light region using a Perkin Elmer Lambda 1050+ spectrophotometer supplied from Pro-Environment Polska Sp. z o.o, Warsaw, Poland.

The chemical composition was investigated using Fourier-transform infrared spectroscopy (FT-IR) using the ATR technique and a SpectrumTwo (PerkinElmer) supplied from Pro-Environment Polska Sp. z o.o., Warsaw, Poland, in the range of 4000–400 cm^−1^, where 32 scans at a resolution of 4 cm^−1^ were performed. Raman spectroscopy, as a complementary approach, was performed using a DXR microscope (Thermo Fisher Scientific, Waltham, Massachusetts, US). Spectra were collected in the range of the Raman shift at 3400–100 cm^−1^, using 532 and 780 nm laser lines, a 10 mm lens, an acquisition time of 5, 10, 20, and 30 s, and 20 repetitions.

The thermal stability of the nanocomposites was assessed by the thermogravimetric method (TGA) using a TG 209 F3 Tarsus apparatus (Netzsch, Frankfurt am Main, Germany). The heating rate was around 10 °C min^−1^ in an open ceramic crucible under a nitrogen atmosphere in the temperature range of 30 to 900 °C.

The glass transition temperature of the materials was estimated using the Differential Scanning Calorimetry (DSC) method using a DSC 204F1 Netzsch device (Selb, Germany). A material sample with an approximate weight of 25 mg was placed in the device’s chamber in a punctured crucible, and the measurement was then taken at the temperature range of 25–220 °C in a nitrogen atmosphere with two heating and cooling cycles. The analysis of the inflexion point of the observed baseline change (T_gInfl_) and the half height of the incremental baseline change (T_gMid_) were used. In order to level the influence of residual solvent, the 2nd heating cycle was analyzed by determining the enthalpy of cold crystallization and melting, T_t._

A thermal stability test of the dynamic mechanical properties was performed on a DMA Artemis device (Netzsch Group, Selb, Germany). The values of the storage modulus (E′) and the loss angle tangent (tan δ) as a function of temperature were determined. The test was performed in the tensile mode (measuring length of 10 mm, sample width of 4 mm, and thickness of 0.38 ± 0.05 mm) with a deformation of 10 μm in the temperature range of −10–135 °C and with a heating rate of 2 °C min^−1^. The deformation was determined with a frequency of 1 Hz. A relatively small amplitude and low deformation frequency allow for measurement in the linear viscoelastic range. 

The tensile mechanical properties were determined in samples with dimensions of 120 mm × 4 mm × 0.38 mm dimensions. The measurement was carried out on a Zwick/Rolel Z010 (Zwick Roell Polska Sp.zoo. Sp.k., Wroclaw, Poland) testing machine at 23 °C. The test speed was 10 mm min^−1^ (1 mm min^−1^ for a modulus). The modulus of elasticity (E*_t_*), maximum stress (σ*_m_*), and deformation at maximum stress (ε*_m_*) were determined.

The capacity to scavenge the free radical DPPH was determined according to the method of Brand-Williams et al. [40]; the scavenging capacity was tested with pure PLA and the composites PLA/HAp and PLA/HAp@CE with a hydroxyapatite and turmeric extract concentration from 1 to 20%, where the HAp was coated with ~10% of CE. Discs of diameter = 10 mm ± 0.05 mm were cut from the materials. Each disc was extracted with 5 mL of 3 different solutions as follows: solution A was ethanol 10% (*v*/*v*), solution B was acetic acid 3% (*w*/*v*), and solution C was ethanol 50% (*v*/*v*). Samples were placed in 50 mL tubes, stopped, sealed, shaken, and then stored in the dark at room temperature for 24 h, 72 h, and 240 h. Obtained extracts (2 mL) were mixed with 2 mL of methanolic solution containing DPPH radicals (0.1 mM L^−1^). The reaction mixture was vortexed thoroughly and left in the dark for 15 min. The absorbance of the mixture was measured spectrophotometrically at 517 nm (Agilent 8453 UV-visible Spectroscopy System, Santa Clara, CA, USA). The radical scavenging activity (RSA) was calculated as a percentage of DPPH discoloration using the equation:

RSA (%) = ((ADPPH − A_S_)/A_DPPH_) ∙ 100% (1)

where A_S_ is the absorbance of the solution when the sample extract is mixed with DPPH methanolic solution and A_DPPH_ is the absorbance of the DPPH solution.

The hydrophilicity of the samples was characterized by measuring the contact angle using a Data Physics OCA 15EC contact angle goniometer (Data Physics Instruments GmbH, Filderstadt, Germany). Measurements were conducted at room temperature, and each sample was measured three times to ensure accuracy. A 2 μL droplet of deionized water was carefully dispensed onto the surface of each sample, which was placed on a microscope glass slide. The contact angle was recorded at various time intervals (1 s, 3 min, 10 min, and until the droplet disappeared) to monitor changes in surface wettability.

To determine the antimicrobial activity of the proposed material, the reference laboratory strain for antidrug testing, *E. coli* ATCC 25922, was used. Tests were conducted on a plate with ~1 cm^2^ square of composites or without samples as a control. Bacteria (5 × 10^5^ CFU per well) were diluted with 5% LB in PBS and incubated with composites for 2 h and 24 h at 37 °C. The number of viable bacteria were enumerated by colony-forming unit (CFU) counting.

## 3. Results and Discussion

### 3.1. Morphology Studies

**Figure 1:**
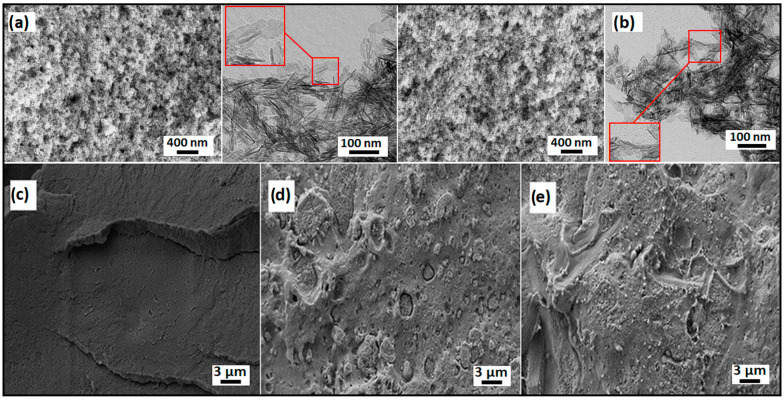
SEM and TEM images of (**a**) HAp and (**b**) HAp@CE and SEM images of (**c**) PLA, (**d**) PLA/5HAp, and (**e**) PLA/5HAp@CE.

Prior to the synthesis of the composite, the morphology of the dried HAp was studied using SEM and TEM. As can be seen in the images below, the HAp is synthesized in a rod-like shape. SEM images reveal the aggregates having an elongated shape, see Figure 1a, while the TEM image seen in Figure 1b confirms the shape of particular nanoparticles are similar to the structures obtained in the literature [41]. Elongated structures were desired for subsequent incorporation into the PLA matrix as the reinforcing filler. Following this, Figure 1c–e show significant changes in SEM images of the cross-section of PLA and nanocomposites, where bare PLA has smoother morphology compared to the samples containing nanofillers. The observed edges in Figure 1d,e indicate the brittle fracture of this material. The cavities that formed after the filler incorporation into the composite can limit the adhesion of the matrix to the filler. The PLA/5HAp@CE image (Figure 1e) revealed a much better dispersion of HAp compared to the samples without CE, and also no filler cavities were observed, indicating improved adhesion between the matrix and the modified filler. This confirms the beneficial effect of using CE as a filler modifier to increase interactions at the filler–polymer interface. The Supplementary Materials (SM) shows more details on the morphology with the different filler contents.

**Figure 2:**
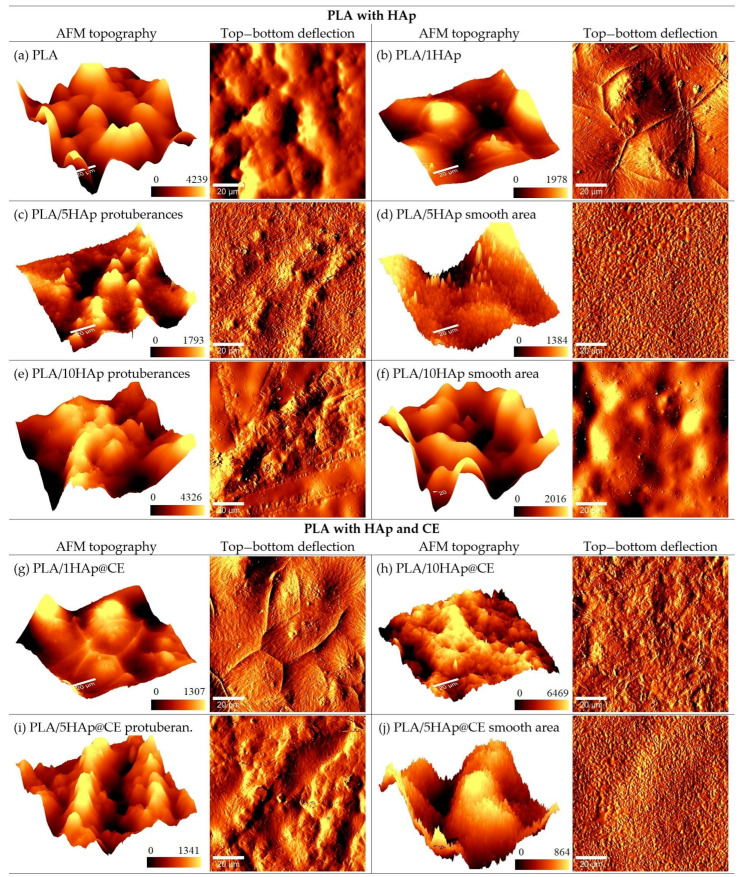
AFM topography and corresponding deflection signal of PLA, PLA with Hap, and PLA with HAp and modified with curcuminoids. Scales on topography figures are shown in nm.

Complementary to the morphology studies with SEM, the topography of the obtained composite was investigated using atomic force microscopy (AFM). Figure 2 presents the AFM topography and top–bottom deflection signal images of PLA, PLA/HAp, and PLA/HAp@CE with different concentrations of HAp. The measurements were performed on the upper side of the polymer formed in the Petri dish. The first and third columns show the topography, and the second and fourth column present the corresponding top–bottom probe deflection signal collected simultaneously, which can reflect local variations in sample height more distinctly than topography. As can be seen in Figure 2a,b, the unmodified PLA has a surface regularly patterned with bubbles and shows no sharp structures. The addition of 1HAp filler changes the surface structure—it becomes smoother and intersects with sharp narrow walls in the valleys between bubbles. The addition of 5HAp causes the sparse emergence of protuberances arranged in the form of spatters on an otherwise relatively smooth area with very small grains. This indicates that the dispersion of hydroxyapatite in the PLA matrix is not uniform on a larger scale. Figure 2c,d show the topography and top–bottom deflection signal of both the smooth area and protuberances of PLA/5HAp. The structure of PLA/10HAp is similar, with protuberances on a relatively smooth area. However, the smooth area does not show grain features, but rather a bubbly surface, similar to pure PLA. This suggests that the composite of PLA and 10HAp is still not uniform. The addition of CE to the PLA/1HAp composite does not significantly change the surface features observed in the PLA/1HAp and PLA/5HAp composites (Figure 2g–i). Both composites, after the CE addition, retain their characteristic attributes. In case of PLA/10HAp@CE (Figure 2j), a higher content of CE causes the spatters to disappear and leaves the surface uniformly rough, but with small grain characteristics, similar to the smooth area of PLA/5HAp or PLA/5HAp@CE rather than the “bubble-like” area of PLA/10HAp. Hence, the addition of the CE modifier to the PLA with a higher HAp content leads to a significantly better dispersion and uniform polymer topography.

Optical microscopy confirmed the quasi-homogenous distribution of CE on the surface, in places other than those of the highest material roughness.

### 3.2. Spectral and Optical Properties

**Figure 3:**
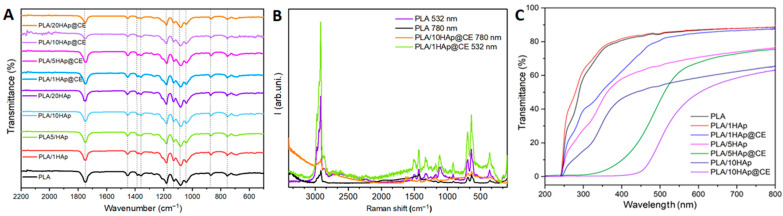
FT-IR (**A**), Raman (**B**), and UV–vis (**C**) spectra of PLA and PLA nanocomposites.

The following analysis using ATR showed only subtle changes in the spectra for the different compositions. The dominant peaks came from the PLA matrix, which was in direct contact with the crystal, so the reflected signal suggests that HAp and CE are embedded in the structure rather than aggregating on the PLA surface. As can be seen in Figure 3A, the main peaks come from vibrations in the PLA, where the peaks can be assigned as follows: 1780 cm^−1^, the symmetric stretching of νC=O in the carbonyl group; 1452 cm^−1^, the asymmetric stretching of δCH_3_ in the methyl group; 1383 cm^−1^, the asymmetric stretching of νCH_3_; 1355 cm^−1^, the symmetric stretching of δCH_3_ in the methyl group; 1180 cm^−1^, the stretching of νCH_3_ in the carbonyl group; 1130 cm^−1^, the stretching of νC-O-; 1080 cm^−1^, the symmetric stretching of νC-O-C in the ether group; 1040 cm^−1^, the bending of OH; 871 cm^−1^, the stretching of νC-COO in the carboxyl group; 755 cm^−1^, the bending of C=O; and 698 cm^−1^, the bending of C-H out-of-plane. The recorded spectrum is in good agreement with data reported in the literature [42,43,44,45]. The presence of the peaks characteristic of the presence of HAp and CE in the composites is barely seen for the dominant PLA signals. These can be distinguished by the complementary spectral analysis.

Thus, Raman spectroscopy (Figure 3B), on the one hand, was used to confirm the FTIR results [46] and, on the other, to provide additional PLA data and evidence of HAp and CE presence. The following bands are diagnostic for the curcumin [47]: 1626 cm^−1^ (carbonyl C=O), 1601 cm^−1^ (aromatic C=C), 1430 cm^−1^ (phenol C-O), 1321 cm^−1^ (C-CH), and 1249 cm^−1^ (C-O). With the PLA/HAp/CE present in the same specimen, bands were highly overlapped or quenched by the luminescence, so the following peaks were the most efficient for monitoring the @CE filler integration: 1630 cm^−1^, 1596 cm^−1^, present only in PLA/10HAp@CE, and the increasing intensity of the ones at 1430 cm^−1^,~1330 cm^−1^, and 1244 cm^−1^.

One can detect the presence of the HAp by a band in the range of 1040–1100 cm^−1^, related to the vibrations in the PO_4_^3−^, and the most typical peak at ~960 cm^−1^ is attributed to the symmetrical stretching of P-O bond, whereas the bending O-P-O are at ~600 cm^−1^ and 440 cm^−1^ (asymmetric and symmetric, respectively).

Finally, PLA exhibits 1180 cm^−1^ and 1090 cm^−1^ peaks typical for the lactic unit (due to the asymmetrical and symmetrical C-O-C stretching), and not specific ~1424 cm-1 band 325 related to the antisymmetrical bending in the CH3 or 1365 cm-1 (δ1CH + δsCH3). Thus, their intensity increases due to the fillers added, which is observed for the PLA/1HAp@CE. The asymmetrical input is dominant. One can distinguish the presence of the HAp (in PLA/1HAp@CE and PLA/10HAp@CE) by a band at ~960 cm^−1^, but it is not possible to determine the crystallinity index of the matrix by either the I_415_/I_400_ (for δCCO vibrations)) or I_960_/I_880_ ratio due to the low intensity of these bands compared to the fillers’ contributions. In the samples in which bands >2920 cm^−1^, typical for the CH_3_ terminal groups, increase respectively to the normalized signal, the fragmentation of chains might be higher in the PLA with added fillers.

Ultraviolet (UV) (100–400 nm) and visible (400–800 nm) transmittance is an essential parameter in for use in packaging design. On the one hand, it is related to protection against photodegradation of the packaged product; on the other hand, photochemical degradation of the polymer can affect the functional properties of the packaging made from it. The properties of the packaging should be preserved until the food product reaches the consumer. In addition, consumers prefer transparent packaging that allows them to check the condition of the food product inside the packaging. It is therefore worth investigating the preparation of advanced materials that meet the requirements for transparency and the UV light barrier [48,49].

Figure 3C shows UV–vis spectra of unmodified PLA and nanocomposites containing up to 10 wt.% fillers. The transmittance of the unmodified matrix material and materials containing 1 wt.% HAp was around 80% and decreased with an increasing filler percentage, reaching a value of around 60% for materials containing 10 wt.% HAp. The applied filler modification using CE did not significantly affect the transmittance of the obtained films in the visible light range. It is worth mentioning that the spectra did not change after the 6 h exposure to the UV–vis light nor to water and 0.1 M NaCl solution for 24 h.

All the materials obtained were impermeable to UV radiation in the range below 250 nm. However, the application of the extract increased the UV barrier range to 300 nm for PLA/5HAp@CE and 400 nm for PLA/10HAp@CE, respectively. The observed effect can be attributed to the property of curcumin contained in the extract, which shows the ability to absorb UV radiation by acting as a natural filter to increase protection against harmful radiation in the obtained materials [50,51]. Materials containing CE therefore show application potential as packaging, especially for light-sensitive materials. They can Protect these materials from harmful UV radiation that can cause oxidative degradation, discoloration, or loss of flavor while maintaining relatively high transmittance in the visible light range.

### 3.3. Thermal Stability

**Figure 4:**
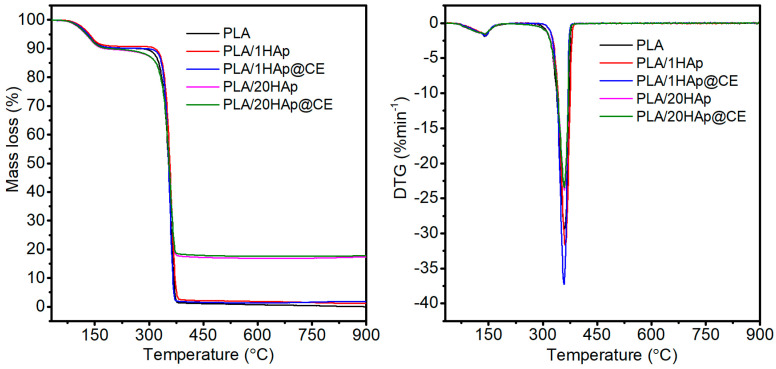
Thermogravimetric analysis of obtained composites and bare PLA.

Subsequently, thermal stability after the PLA modification was investigated using thermogravimetry (TGA). Figure 4 shows example thermograms of the tested materials. The decomposition of PLA and composites occurred in two stages. The first stage of mass change in the temperature range of 80–150 °C is associated with the evaporation of residual solvent from the polymer films obtained by the solvent method, which was also noted in the work [52].

The second stage of mass loss is correlated with the decomposition of the polymer matrix and occurs in the temperature range of 300–400 °C with a maximum decomposition rate at T_DTG_ 358–363 °C. These results are consistent with the literature [53,54].

Table 1 shows the results of the thermal stability test performed on all the materials produced. The amount of solvent in the tested materials determined from DTG was around 10%. Therefore, due to the presence of solvent, in order to determine the effect of used modifiers on the thermal stability of the PLA matrix, the extrapolated value of the temperature of the beginning of the change in the weight of the sample in terms of polymer decomposition (T_onset_) was analyzed. Composites containing up to 10 wt.% HAp with and without CE coating in the matrix were characterized by a higher T_onset_ value compared to unmodified PLA. The observed increase in thermal stability at a negligible proportion of the filler may be due to homogeneous optimal dispersion of the HAP filler in the PLA matrix, and thus uniform heat dissipation in the composite system. Moreover, appropriate uniform dispersion may form a physical barrier and slow down the decomposition of PLA macromolecules [55].

For the addition of 20% of HAp with and without CE coating, these values are slightly lower, by 1.2 and 0.3 °C. Our results are in agreement with the studies presented in [52], where lower thermal stability results were also obtained for the PLA/20Hap composites compared to unmodified PLA. This may be the effect of accelerated heat transport to PLA macromolecules by a significant proportion of the mineral filler with a higher thermal conductivity and, thus, a slight acceleration of matrix degradation. In addition, with a higher proportion of filler in the matrix, agglomerates of the filler are formed, which can locally overheat PLA macromolecules and thus lead to their faster decomposition in the initial phase. A similar description of the phenomenon was proposed in [56], which focused on the modification of PCL by HAp. The decrease in thermal stability was also observed with a significant proportion of filler, suggesting that a poor dispersion of filler in the matrix lowers the activation energy barrier and hence leads to early degradation. As the amount of filler in the polymer matrix increases, filler tends to aggregate, affecting the final properties of the polymer, including thermal stability [57]. Moreover, other studies suggest that calcium phosphates such as HAp, especially with a significant proportion in the matrix, show a tendency to induce polymer chain scission, which also reduces the thermal stability of composites [56].

Slightly higher T_DTG_ values characterize all of the composites compared to the unmodified matrix. The highest value was recorded for the composite containing the lowest HAp content and a lack of CE. The addition of fillers slightly affected the shift of T_DTG_ towards a higher value, which may suggest a slightly beneficial effect of the fillers used. The filler modification used did not affect the T_DTG_ value of the composites.

**Figure 5:**
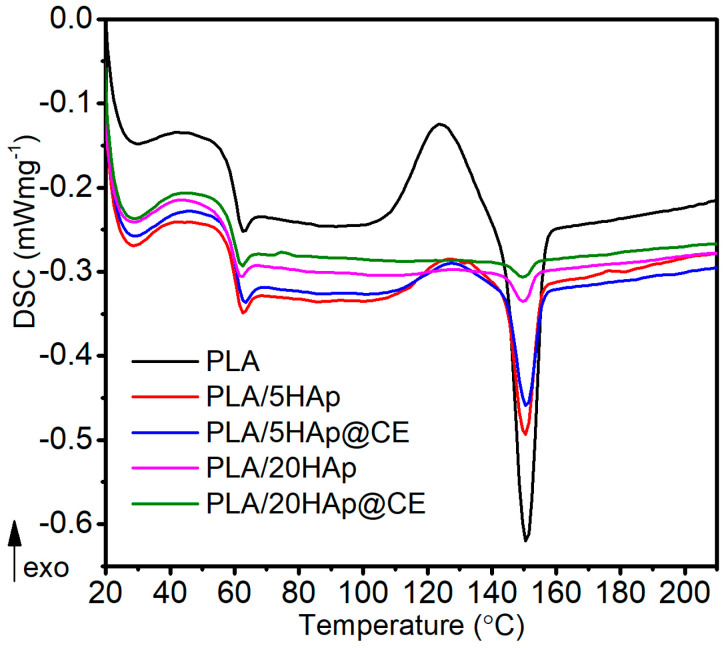
DSC curves.

Next, the obtained materials were investigated using DSC and DMTA techniques. DSC curves of selected nanocomposites are shown in Figure 5 and the Supplementary Materials. The values of glass transition temperature T_gMid_ and T_gInfl_ determined by the DSC method during the second heating cycle of PLA and the composites are similar, regardless of the amount of filler and its modification method, and are in the range of 58.3–59.6 °C and 59.7–60.6 °C, respectively. Based on this, it can be suggested that there is no significant effect of the amount and type of filler on changes in the amorphous region of PLA. Also, the plasticizing effect of HAp modified with turmeric extract on the PLA matrix at higher filler concentrations compared to the T_g_ results obtained by the DMA method was not confirmed.

It was also observed that on DSC thermograms of samples containing up to 10% HAp there is an exothermic peak associated with the cold crystallization process, and the area of this peak decreases with increasing filler content and is no longer observed at the maximum HAp content. This peak is due to macromolecular reorganizations in the amorphous domains, which again gain increased mobility during the DSC heating process [58,59]. The addition of filler decreases the composite’s permeability by reducing the macromolecular segments’ mobility in the amorphous region in the cold crystallization range [60,61].

The melting point value is also comparable regardless of the sample composition (approx. 150 °C), and the difference is approx. 1 °C. There is a subtle decrease in the value determined according to the methodology [62,63] and the degree of crystallinity (K). However, the type of PLA used, i.e., 2003D, contains approximately 4.3% D-Lactide and has a lower crystallization ability [64], indicating the amorphous nature of the PLA films. The results on DSC analysis are presented in Table 2.

**Figure 6:**
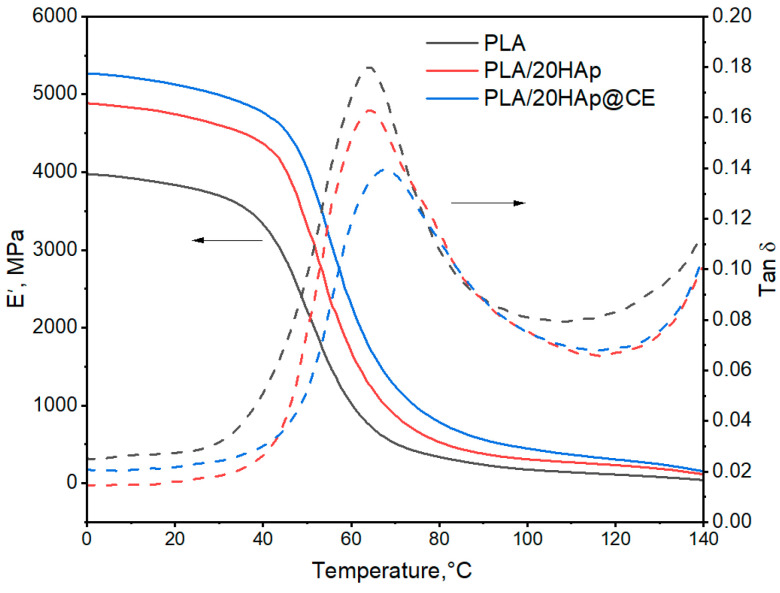
DMA thermograms of PLA and nanocomposites containing 20% filler.

Figure 6 shows exemplary DMA thermograms (storage modulus (E′) and Tan δ) of the selected materials, while Table 3 summarizes the readings of the E′ modulus at 0 and 25 °C and the values of the glass transition are analyzed, which were determined from an extrapolated value from curve E′ (E′_onset_) and maximum Tan δ.

From the obtained DMA curves, the values of the E′ modulus at 0 °C and 25 °C temperatures were read (Table 3). Compared to unmodified PLA, the E′ modulus values of the composites are higher and increase with increasing filler content in the matrix regardless of the type of modifier used. The highest E′ values are characterized by composites containing the maximum proportion of filler. These values at 25 °C were higher than those of unmodified PLA by 24% and 31% using 20HAp and 20HAp@CE, respectively. When HAp@CE was used, higher E′ modulus values of up to 5% were observed compared to composites with HAp, which could be related to a more favorable dispersion of the modified filler in the PLA matrix. Increasing the proportion of HAp@CE in the matrix resulted in slightly lower values of the E′ modulus, which may be due to the use of CE extract and may be a result of its plasticizing effect towards the PLA matrix [65,66,67]. Oily substances are also present in the extract [42], which can plasticize the PLA matrix. In the materials containing the highest percentages (HAp@CE), maximum-level E′ values were obtained, regardless of the filler, by extract modification. This effect was related to the improved dispersion of HAp in the PLA matrix regardless of the plasticizing effect by CE. 

The reduced T_g_ values of unfilled PLA compared to the literature data [68,69] may indicate a plasticizing effect of residual solvent in the composite. Nevertheless, TGA analysis showed the same solvent content in the matrix regardless of the composite type. Therefore, it can be concluded that the observed changes in T_g_ values are due to PLA macromolecule–filler interactions. 

The introduction of unmodified HAp into the PLA matrix causes a slight increase in the T_g_ value read from the E′_onset_ point. A T_g_ value higher by around 3 °C compared to PLA was obtained for the PLA/20HAp composite. As the filler content increases, the T_g_ value increases, which may be due to the restriction of segmental movement by the filler and the shift of PLA segmental response towards a higher temperature value. Composites containing modified filler up to 10% show a lower T_g_ value compared to PLA. Unmodified HAp increases the T_g_ value of the matrix of the composites more than HAp@CE, which may confirm the slight plasticizing effect of CE extract on the polymer matrix. However, the highest value was obtained for PLA/20HAp@CE and was 4.4 °C higher than for PLA. A similar trend was observed when analyzing the T_g_ value read from the tan δ maximum. This effect is related, as in the case of the conservative modulus, to the significant proportion of mineral filler. However, unambiguous determination of the plasticizing effect of CE on the PLA matrix using the DMA method is difficult due to the presence of residual solvent in the samples. In the DSC studies, no significant change in T_g_ was observed in the nanocomposites relative to the matrix material.

### 3.4. Mechanical Studies

As the role of the curcumin extract in the nanocomposite was the improvement of the HAp dispersion in the material, it could also change the mechanical properties of the final product. Therefore, mechanical studies were performed to provide information on the elasticity of the PLA/HAp and PLA/HAp@CE composites. Table 4 summarizes the results of the tests of the mechanical properties in static tension. The analysis showed an increase in the modulus of elasticity in meta-materials with HAp compared to unmodified PLA. However, its values are dependent on the dispersion of the filler in the matrix as indicated by the variability of the results in materials containing 10 and 20% HAp [21,22,23,70]. CE modification reduced the *E_t_* values of nanocomposites with HAp@CE compared to materials with unmodified hydroxyapatite, which may confirm the plasticizing effect of the extract on the matrix [71].

**Figure 7:**
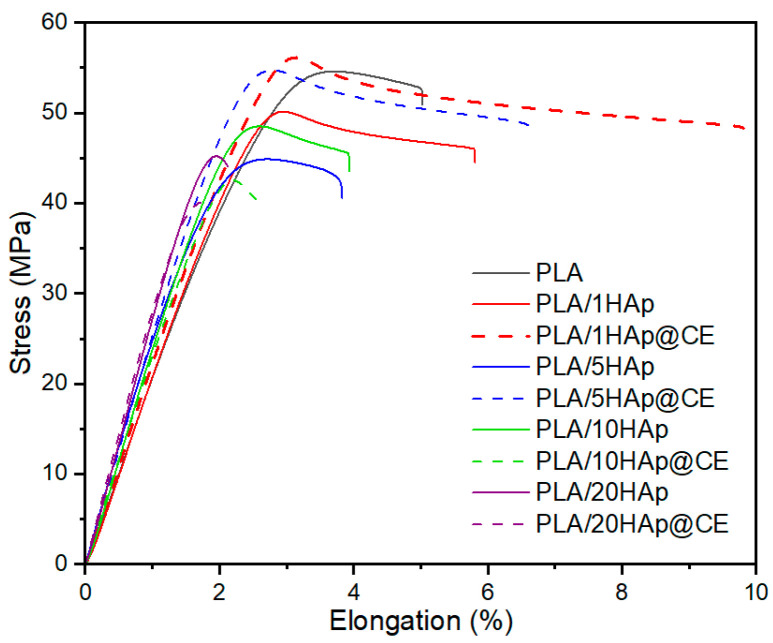
Results of the stretching of the composite.

Similar conclusions can be drawn from the analysis of elongation at tensile strength, where a significant increase was observed in materials containing up to 5% HAp@CE in comparison with the PLA/HAp composites. The use of CE as a modifier had a positive effect on tensile strength. Materials containing 1 and 5% HAp with extract were characterized by a value of this parameter similar to unmodified PLA, while unmodified HAp caused deterioration of the values of *σ_m_.* It is known that the improvement of the mechanical properties of PLA with the use of HAp depends on its dispersion in the matrix and the structural parameters of the filler itself. The analysis showed that the application of CE can favorably affect the dispersion of hydroxyapatite, which was confirmed by SEM observations. In addition, the results indicate a favorable effect of the extract on improving the mechanical properties of the PLA/HAp composite with a small amount of mineral filler, which may be useful in applications of this composite in the packaging, coating, biotechnology, and/or engineering, including biomedical engineering. A higher content like 10% and 20% worsens the mechanical properties, making the material brittle. Figure 7 shows the effect of curcuminoids on the resistance to stretching of the composite. However, the effect of curcuminoids on the mechanical properties of PLA needs further investigation, as also indicated by mutually exclusive literature reports where, on the one hand, an improvement in tensile strength with a simultaneous decrease in elastic modulus after the introduction of curcumin into PLA films was observed [72]. On the other hand, its plasticizing effect was indicated [71], and no improvement in tensile properties was shown [34].

### 3.5. Capacity to Scavenge the Free Radicals

**Figure 8:**
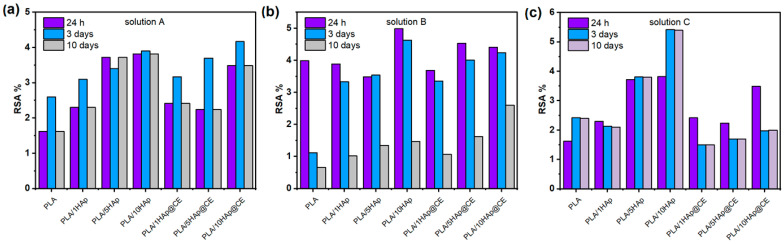
(**a**) RSA of PLA composites extracted in solution A (ethanol 10%), (**b**) RSA of PLA composites extracted in solution B (acetic acid 3%), and (**c**) RSA of PLA composites extracted in solution C (ethanol 50%).

The proposed material has the potential to be used in the agri-food fields, so the following analysis was based on the evaluation of the antioxidative properties of the material. Based on the thermal stability and mechanical studies, the samples containing 20% wt% of the filler in the PLA matrix were excluded from the following studies for the worsening of the thermal stability as well as the mechanical properties. The studies were performed in different media named solutions A (ethanol 10%), B (acetic acid 3%), and C (ethanol 50%), and it can be observed that longer storage time enhances the radicals scavenging activity (RSA). The highest antioxidant capacity was detected in materials with solution C, see Figure 8. An acidic environment, which characterized solution B, made the extraction slower and the RSA of the materials increased gradually during the storage (Figure 8b). Unexpectedly, the PLA/HAp composites have a higher RSA than the PLA/Hap@CE composites, even though curcumin extract has a great antioxidant activity [73]. This is caused by the degradation of PLA, which is susceptible to hydrolytic degradation in the presence of water. During the PLA immersion in water solution, the release of lactic acid (LA) can be observed [74]. Since PLA is a hydrogen donor it can react with DPPH* radicals. Hydroxyapatite is a porous material and its surface increases in an aqueous media for the solvatation. This explains why a higher concentration of HAp in PLA composites gives higher RSA values. In the case of the PLA/HAp@CE composites, even if CE has a great RSA itself, we can assume that active compounds of curcumin react against PLA degradation first, and then second with DPPH*. The hydrolysis of PLA was accelerated in 50% ethanol, which is related to the highest release of LA. This observation was confirmed in research by Iniguez-Franco et al. [74].

### 3.6. Contact Angle Studies

**Figure 9:**
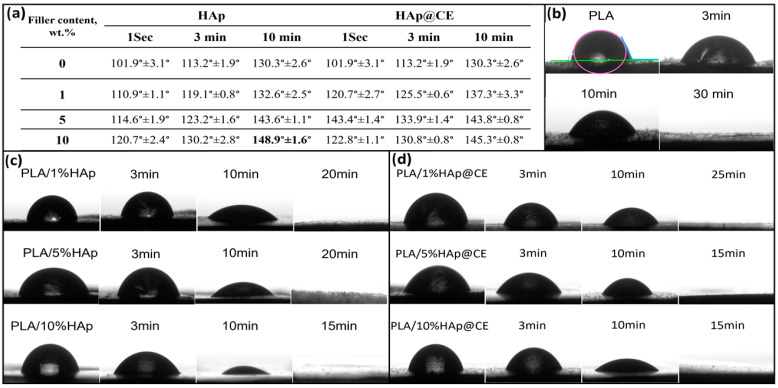
(**a**) Table of nanocomposites’ hydrophilicity measurements: (**b**) PLA, (**c**) PLA and 1, 5, 10% HAp extracted, and (**d**) PLA composites extracted (1, 5, 10% of HAp@CE).

Hydrophilic fillers are widely recognized for their ability to alter the properties of thermoplastic and hydrophobic polymers to create hydrophilic composites [75]. The hydrophobic characteristics of PLA stem from the presence of large, non-polar methyl groups (–CH_3_) and non-polar C-H bonds, with minimal polar groups like carboxyl (–COOH) contributing to its surface properties [42]. However, incorporating HAp as a bio-filler can notably enhance the hydrophilicity of PLA composites. HAp, a natural mineral form of calcium apatite, features hydroxyl (–OH) and phosphate groups that interact with water molecules, thereby increasing the overall hydrophilicity of the composite material [76,77]. As illustrated in Figure 8a, a water droplet placed on the surface of pure PLA exhibited a contact angle of 101.9 ± 3.1°, which completely disappeared after 30 min. In contrast, composites containing different proportions of HAp demonstrated significant water absorption due to the abundance of hydrophilic hydroxyl groups in HAp. Specifically, the contact angle of the modified nanocomposite PLA/HAp and PLA/HAp@CE increased to 120.7° ± 2.4° and 122.8° ± 1.1°, respectively (Figure 9).

### 3.7. Antimicrobial Activity

**Figure 10:**
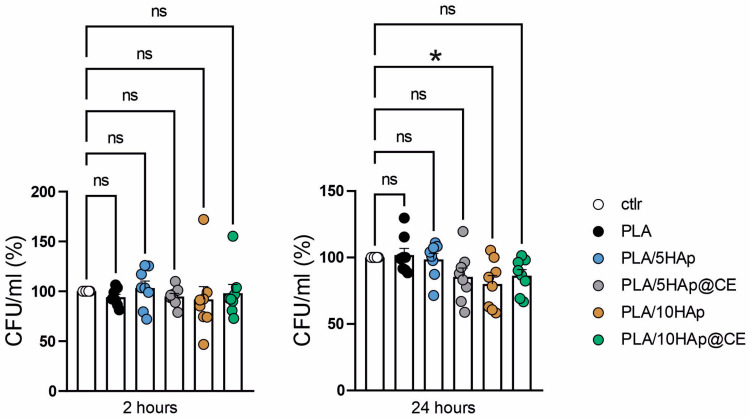
Bacteria viability in CFU mL^−1^, shown as the percentage of control cells. ns, * *p* < 0.05, one way ANOVA, Sidak’s multiple comparisons test, n = 8, pooled from 4 independent experiments.

Next, the composites were studied in terms of their antimicrobial activity against *E. coli*. Incubation time with composites was performed for 2 and 24 h and the attached graphs in Figure 10 show the average cumulative results from four biological replicates, each with two technical replicates. After 2 h, none of the tested composites showed antimicrobial activity when compared to the control group. However, after 24 h, we observed a significant antibacterial effect for the PLA/10Hap (*p* < 0.05), as well as promising trends for the PLA/5HAp@CE and PLA/10HAp@CE composites. Our results align with studies that confirm that hydrophilic, modified HAp composites can exhibit antibacterial properties over extended incubation periods, whereas hydrophobic composites demonstrate stronger bactericidal properties over shorter incubation periods [75,76]. In cases where no antibacterial effect was observed, we found that there was no increased bacterial growth compared to the control without the composite. This suggests that bacteria have a weak adherence to these composites. Given that HAp is a porous material with hydrophilic properties that readily attract bacteria [75], we believe our modifications to HAp successfully minimized this effect to a certain extent. However, the specific impact of PLA/HAp on bacterial adhesion needs further investigation. Considering these findings, our PLA/HAp composites offer desirable features for various material applications, particularly in food packaging, due to their bacteriostatic properties.

## 4. Conclusions

This work demonstrates a bioinspired composite containing biocompatible PLA as a polymer matrix filled with HAp and turmeric extract, chosen as a dispersing agent to protect HAp from aggregation in the composite and for its high thermal stability. The incorporation of the nanostructured filler and plant extract was carried out as an alternative to commercial plastics, addressing a deep need to provide non-cytotoxic plastic modifiers for use in many fields. As HAp offers a well-developed surface, it can be easily coated with various chemicals, such as natural plant extracts, to replace synthetic binders in composites. Here, PLA was successfully filled with 1, 5, and 10% HAp and plasticized with curcumin extract. Immobilization of the curcumin extract improved the mechanical and antioxidative properties of the composite, and enhanced the optical properties, allowing the proposed material to be used in the food industry. Furthermore, the inclusion of a higher HAp content and curcumin into the PLA matrix amplified the bacteriostatic capabilities of the composites. The properties of the proposed composite were shown to depend on the HAp and CE content. The proposed material has the potential to be used in agri-food, engineering, biotechnology, and/or even biomedical applications.

## Figures and Tables

**Table 1:** Results for thermal stability.

Sample	T_onset_, °C	T_DTG_, °C
PLA	339.0	358.4
PLA/1HAp	344.3	363.5
PLA/5HAp	343.5	361.8
PLA/10HAp	341.4	361.3
PLA/20HAp	337.8	359.4
PLA/1HAp@CE	342.9	359.2
PLA/5HAp@CE	343.7	362.0
PLA/10HAp@CE	342.2	361.6
PLA/20HAp@CE	338.7	359.8

**Table 2:** DSC analysis results.

Sample	T_gMID_, °C	T_gINFL_, °C	ΔH_cc_, J g^−1^	ΔHm, J g^−1^	T_t_, °C	K, %
PLA	59.3	60.3	14.12	17.27	150.9	3.4
PLA/1HAp	58.9	60.5	13.46	16.82	150.3	3.6
PLA/5HAp	59.3	60.5	4.42	7.24	150.4	3.2
PLA/10HAp	59.1	60.6	0.92	3.70	150.3	3.3
PLA/20HAp	59.2	60.2	0.05	1.15	149.8	1.5
PLA/1HAp@CE	58.5	59.8	19.0	22.21	149.7	3.5
PLA/5HAp@CE	59.6	60.7	3.62	5.82	150.7	2.5
PLA/10HAp@CE	59.0	60.6	1.60	3.80	150.1	2.7
PLA/20HAp@CE	58.3	59.7	0	1.61	149.8	2.1

**Table 3:** DMA analysis.

Filler Content, wt.%	E′, MPa		T_g_, °C	
	0 °C	25 °C	E’_onset_	tanδ
PLA	3923	3754	40.2	64.3
PLA/1HAp	4084	3885	39.8	63.0
PLA/5HAp	4101	3946	41.4	65.6
PLA/10HAp	4718	4494	41.6	63.5
PLA/20HAp	4885	4707	43.6	64.4
PLA/1HAp@CE	4211	4039	40.0	63.0
PLA/5HAp@CE	4331	4105	38.7	61.8
PLA/10HAp@CE	4611	4357	37.0	60.7
PLA/20HAp@CE	5149	4969	44.6	66.3

**Table 4:** Mechanical properties.

Filler Content, wt.%	HAp			HAp@CE		
	*E_t_*, MPa	*σ_m_*, MPa	*σ_m_*, %	*E_t_*, MPa	*σ_m_*, MPa	*σ_m_*, %
0	2200 ± 132	54.7 ± 1.5	3.2 ± 0.5	2200 ± 132	54.7 ± 1.5	3.2 ± 0.5
1	2120 ± 21	49.9 ± 0.8	2.9 ± 0.02	2160 ± 35	55.5 ± 0.8	3.2 ± 0.1
5	2610 ± 32	44.6 ± 0.4	2.6 ± 0.2	2490 ± 56	54.9 ± 0.4	5.8 ± 0.9
10	2390 ± 65	48.2 ± 3.0	2.6 ± 0.1	2250 ± 72	43.2 ± 5.8	2.5 ± 0.3
20	2560 ± 110	45.2 ± 1.6	2.0 ± 0.1	2890 ± 75	40.9 ± 1.7	2.1 ± 0.3

## Data Availability

The data supporting this study’s findings are available from the corresponding authors (Magdalena Osial, Sławomir Wilczewski) on request.

## Supplementary Materials

The following supporting information can be downloaded at: https://www.mdpi.com/article/10.3390/polym16152169/s1, Figure S1: DSC curves of PLA/1HAp, PLA/1HAp@CE, PLA/10HAp, PLA/10 HAp@CE; Figure S2: SEM images of PLA/1HAp, PLA/1HAp@CE, PLA/20HAp, PLA/20 HAp@CE.
