# Supplementary material for: Incorporation of Nanostructural Hydroxyapatite and Curcumin Extract from Curcuma longa L. Rhizome into Polylactide to Obtain Green Composite
Source: Polymers (Basel). 2024 Jul 30;16(15):2169. doi: 10.3390/polym16152169 (PMC11315054; doi:10.3390/polym16152169)
Supplement: Supplementary file 1 [file polymers-16-02169-s001.zip › polymers-3075102-supplementary.pdf]

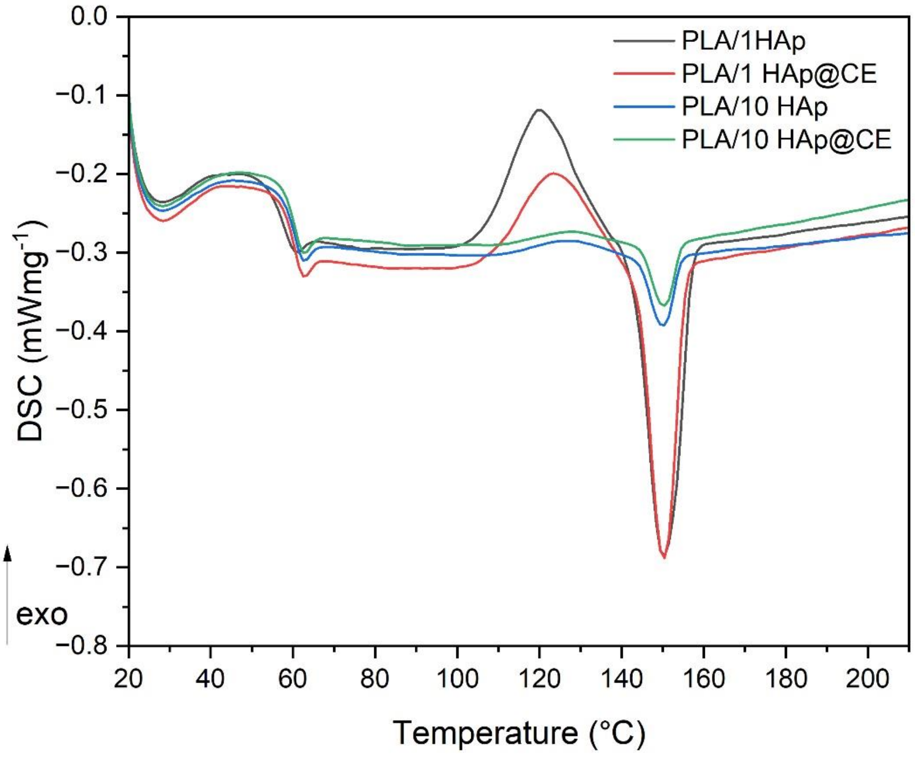

Figure S1. DSC curves of PLA/1HAp, PLA/1HAp@CE, PLA/10HAp, PLA/10 HAp@CE.

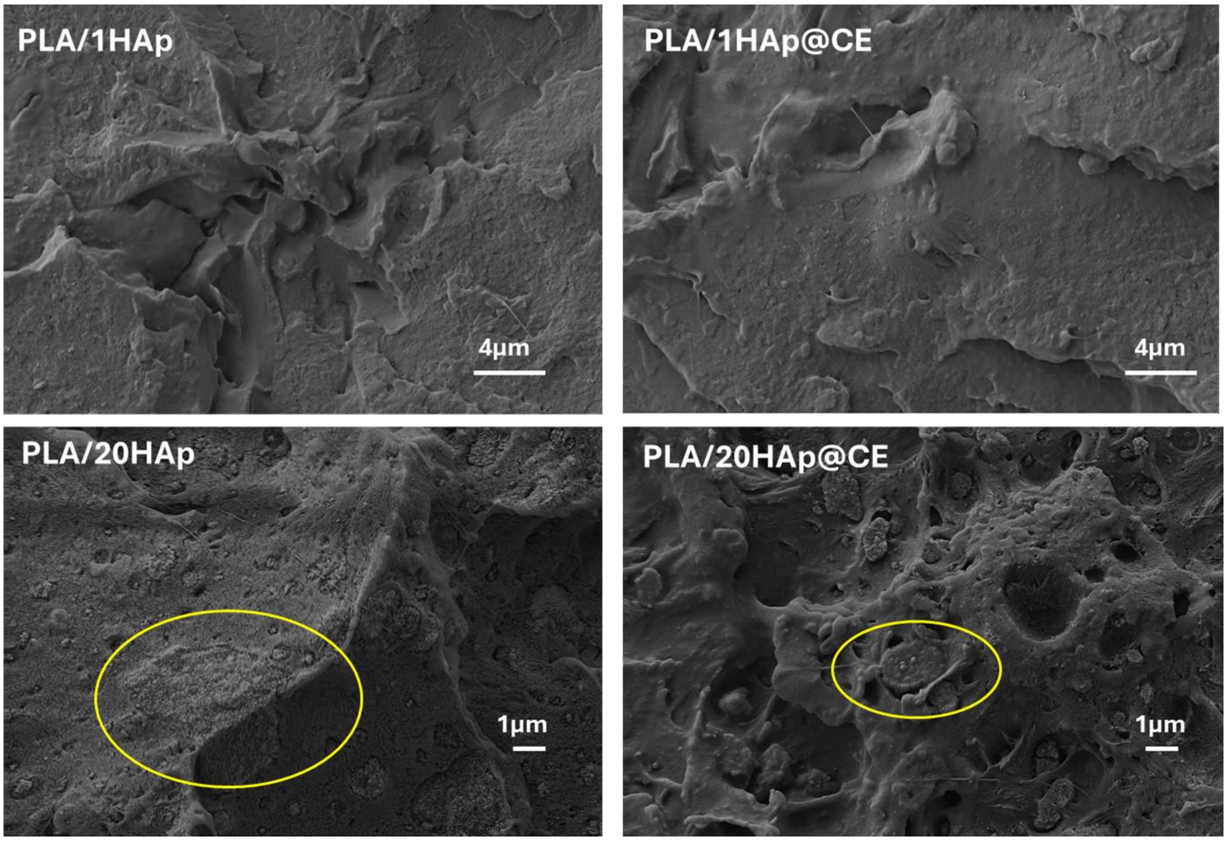

Figure S2. SEM images of PLA/1HAp, PLA/1HAp@CE, PLA/20HAp, PLA/20 HAp@CE
